# Building on health care access for children in Spanish-language settings

**DOI:** 10.1093/haschl/qxaf076

**Published:** 2025-07-24

**Authors:** Rishika Selvakumar

**Affiliations:** Faculty of Graduate & Postdoctoral Studies, University of British Columbia, Vancouver, Bc, Canada, V6T 1Z2

Dear Editor,

Zaylskie et al^1^ provides insightful data on the access and disparities in health care within English- and Spanish-speaking households in the United States. This nationally representative survey emphasizes the effects of primary household languages on health care utilization among children. However, increased research would be instrumental to analyze age-group differences (ie, between 0 and 5 years, 6 and 12 years, and 13 and 17 years). As highlighted by the authors as a limitation, the level of English fluency as a factor to impact health disparities requires continued investigation. Further data collection, especially within smaller, local settings instead of a national setting, to assess the differences in parental and child fluency and the influence on patient–provider relationships is needed. This research would delineate whether gaps in health access are found due to language barriers or systemic bias.

Recognizing that this study is based on data collected in the 2021 National Survey of Children's Health, when COVID-19 was still a prominent concern, promising research opportunities exist by exploring the social changes since the pandemic and the subsequent shift in health disparities. Other geographic and community factors are also important to consider, particularly the impacts of a strong cultural community on health care service access and outcomes.

Another valuable asset in research would be the investigation of whether similar health disparities exist for children in other visible minority groups where English is not the dominant language. Zaylskie et al^1^ suggest the implementation of policies on improving language translation and cultural humility education. These ideas could be further developed by including patient–provider perspectives and assessment by geographical area.

This study significantly contributes to understanding the underlying factors impacting health care access in visible minority communities. Additional research with consideration of age range, different languages, and implementation and assessment of proposed policies would actively enhance current standards of care.

## Supplementary Material

qxaf076_Supplementary_Data
